# Joint Communication and Radar Sensing: RF Hardware Opportunities and Challenges—A Circuits and Systems Perspective

**DOI:** 10.3390/s23187673

**Published:** 2023-09-05

**Authors:** Padmanava Sen, Armen Harutyunyan, Muhammad Umar, Shahanawaz Kamal

**Affiliations:** Barkhausen Institut, 01187 Dresden, Germany; armen.harutyunyan@barkhauseninstitut.org (A.H.); muhammad.umar@barkhauseninstitut.org (M.U.); shahanawaz.kamal@barkhauseninstitut.org (S.K.)

**Keywords:** communication, radar, joint communication and radar sensing, RF, antenna, 6G, front-end, mmWave, hardware

## Abstract

This paper focuses on the topic of joint communication and radar sensing (JCRS) and its applications in the scope of upcoming sixth-generation (6G) technology. While the fifth-generation applications have reached the consumer market in the last few years, JCRS has been identified as one of the key technologies for next generation networks. The role of JCRS will vary, ranging from tasks such as radar coordination, context awareness for communication, enhanced security, increased availability, and improving the resilience and trustworthiness of future networks. In this work, the niche of JCRS technology in the future 6G ecosystem, as well as several potential applications, are discussed with a focus on RF hardware. The use of centimeter (cmWave) and millimeter (mmWave) frequency spectrums in the context of JCRS system implementation have been further elaborated. After presenting the near-term application scenarios, the circuit implementation perspectives are investigated in terms of radio frequency (RF) front-end architectures, antenna implementation, and phased arrays. Different communication and radar antenna options are compared, and the best candidates are identified. The packaging options are also presented. From circuit and system perspectives, link budget and self-interference cancellation (SIC) are highlighted. Furthermore, future directions including the next steps on the path to enabling JCRS technology are presented throughout this article. Prior works focused more on physical layers and network capabilities of JCRS systems, with less focus on hardware possibilities; to fill this gap, this article aims to contribute to this exciting research topic with a holistic review of RF hardware, highlighting the diversity of applications and the available technologies to tackle the near- and long-term needs of consumer applications.

## 1. Introduction

In recent years, the interest to bridge the gap between communication and sensing systems has increased substantially in the academic and industrial community [1,2,3]. The introduction of mmWave bands in cellular systems reduced the difference in the frequency of operations and, as a consequence, the hardware used. At the same time, as use cases evolve, specifically those targeting 6G networks [4,5], the is an increasing need to use electromagnetic (EM) waves in sensing, for instance in radar sensing applications, in order to be part of the communications ecosystem. While this idea may enable radar-as-a-service (RaaS), there are still several standardization bottlenecks. The immediate benefits, such as the flexible and efficient use of radio sources, are clearly visible for the JCRS system; however, it is yet to be seen how it attracts the communication infrastructure and radar providers. As of today, research communities have reached a consensus that, in terms of usage, JCRS has the potential to revolutionize existing applications, from connected driving to mobile communications, with the addition of context awareness. It could also enable new applications in medical devices, secure Internet of Things (IoT), and the Industry 4.0 domain [6]. 

With the introduction of 3GPP Rel. 16, new advanced localization capabilities utilizing time of arrival (ToA), time difference of arrival (TDoA), and angle of arrival (AoA) positioning methods have emerged. In addition, modern advanced driver assistance systems (ADAS) utilize communication capabilities that were standardized in 3GPP Rel. 15 vehicle-to-everything (V2X) technology. However, future JCRS systems will require new levels of co-existence, sensor fusion, and real-time positioning features powered by machine learning, as well as enhanced privacy capabilities. JCRS technology is anticipated to support various 6G verticals, such as smart cities, the automotive industry, healthcare, manufacturing, and the likes. The numerous use cases that would leverage efficient spectrum usage, energy efficiency, and resilience of the future verticals are as follows:

- Communication-assisted radar: These are advanced, next-generation radar systems that utilize communication with other vehicular devices to increase traffic awareness, particularly improving the accuracy of pedestrian detection and infrastructure locations. However, additional measures are necessary to increase data security, thus preventing unauthorized access to vast amounts of sensitive data.
- Radar-assisted communication: The concept utilizes the existing communication and radar infrastructure to improve ecosystem characteristics, which will require huge standardization efforts.
  ▪ Spectrum efficiency could be enhanced by sharing the operating frequency. With the introduction of frequency range (FR2) bands for the fifth-generation (5G) network, the large bandwidth can be utilized to enable the co-existence of radar communication.
  ▪ Based on sensing information, adaptive systems can be implemented by adjusting the power level and beamformer angle, thus improving the energy requirements.
  ▪ Adding sensing data such as the angle of arrival or time of flight to existing 5G localization algorithms can improve the positioning accuracy.
- Radar as a Service: RaaS is an emerging concept in which sensing information is provided to interested agents to increase the efficiency of the provided services. This includes providing real-time traffic information to smart city monitoring centers or providing weather monitoring information, such as precipitation areas, to weather agencies. Other aspects include providing real-time 3D maps of the environment for creating digital twins of infrastructure objects for interested parties. As communication is already used as a service in today’s networks, RaaS along with communication as a service can enable the flexible use of resources, depending on the context. This will further increase the demand and usage of JCRS devices in the future.

Overall, the introduction of joint communication and sensing technologies will increase the resilience of sensing services. The sensor fusion and the addition of sensing redundancy/diversity will mitigate the errors occurring due to abrupt changes in environment and communication channels. However, every system will not function as a co-designed system. Depending on the needs and feasibility of the hardware, the nature of integration between communication and radar systems will vary. In some cases, tight collaboration at a software level will be sufficient, whereas a co-designed and shared RF hardware will be a necessary requirement in other cases [6]. However, it goes without saying that the feasibility of RF hardware that can address the needs of both functionalities will play an important role in the deployment of user devices. This paper focuses more on the RF hardware usage in the field of JCRS. JCRS is considered a subset of the field of Joint Communication and Sensing (JC&S), sometimes referred to as Integrated Sensing and Communications (ISAC). There can be different sensing methodologies other than radars. However, these alternative sensing methods (e.g., ultrasonic) are outside the scope of this paper. As the co-design opportunities are higher at mmWave frequencies, particular emphasis has been given to mmWave frequencies beyond 30 GHz and the bands close to this (e.g., 5G new radio (NR) band at 24–28 GHz).

In Section 2, we dive deeper into the application scenarios and key enablers for RF hardware. In Section 3, we touch on the circuit level perspectives for JCRS front-ends, along with the antenna domain, beamforming (BF) enablement, and packaging challenges. In Section 4, a system-level viewpoint is presented, including the system validation approaches needed for JCRS enablement. Section 5 concludes this paper. 

## 2. Application Scenarios and Key Concepts/Enablers for RF Hardware in JCRS

In this section, the needs for communication-assisted radars and radar-assisted communications scenarios are further illustrated, along with the implications for RF hardware requirements. Different use cases exist in automobile, mobile, Industry 4.0, virtual/augmented reality (VR/AR), robotics, IoT, and other evolving applications. However, not every application requires a completely new RF hardware. Some applications may require a modified front-end, some an updated antenna structure, and some may require completely co-designed RF hardware. Some applications may even work with existing hardware and will require only an update in software to enable the sensing capability. To summarize, there will be different levels of integration. The authors of [7] envisioned the levels of integration into four different types of systems—isolation, coexistence, cooperation, and co-design. In this case, isolation means no interaction between these two systems, while co-design is the most integrated and tightly coupled system. While co-design is the most desired approach to obtain the most out of hardware reuse [8], the immediate use cases may greatly benefit from coexistence and cooperation approaches. The challenge of standardization in the radar domain and the privacy aspects of sensing in multiple use cases may also limit usage. However, the use of passive radars to aid in communication can be implemented possibly with limited efforts [9]. At the same time, the sharing of information with a cooperation approach can be done using a system tied at higher layers (e.g., software). In general, coexistent systems may work in different bands, whereas cooperation may share the spectrum, but with separate hardware. The co-designed approach aims to share both spectrum and RF hardware. The extensive usage of hardware that can address both of the systems’ requirements can lead to ubiquitous sensing along with ubiquitous communications, thus making radars cheaply available and RaaS a reality in consumer applications.

### 2.1. Scenarios

There are several scenarios that can be considered while envisioning the widespread usage of JCRS. The authors of [10] described communication challenges across several verticals of future communication systems. Numerous scenarios can potentially reuse the existing infrastructure/hardware benefiting from JCRS technology. Here, three such scenarios are discussed, placing a focus on modem system requirements.

#### 2.1.1. Traffic Monitoring

The combination of communication and sensing information extracted from different sources and further data processing can provide a real-time reliable picture of traffic condition; hence, it can improve transportation management and public safety. In this scenario, the data collected from different sensors (e.g., radars) are communicated to the central processing unit or to a cloud-based system. The system then can interactively adjust traffic control parameters, such as traffic signals or dynamic lane management settings, based on traffic congestion levels, vehicle counts, average speeds, and traffic flow patterns [11].

**Figure 1:**
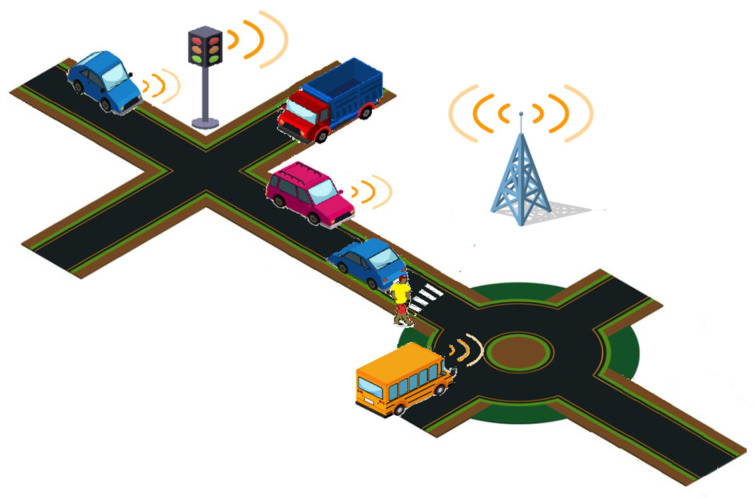
Traffic monitoring scenario.

With a reliable traffic monitoring system in place, incidents such as accidents or road closures can be detected in a timely manner and communicated to the relevant authorities, which will enable faster emergency response, thus, improving public safety and reducing response times. This aligns with the broader concept of smart cities. By integrating traffic data with other services, such as public transportation systems, parking management, and environmental monitoring, cities can enhance their overall efficiency and improve transportation networks. In combination with deep learning, big data can be used to implement predicative traffic control based on traffic monitoring patterns. This scenario is illustrated in Figure 1.

#### 2.1.2. 3D Mapping

Three-dimensional map generation using JCRS is an advanced and innovative approach that leverages both communication and sensing technologies to create detailed three-dimensional (3D) maps of outdoor and indoor environments. Among other technologies, this concept uses the reflection from the existing communication channels to improve map accuracy, create interactive digital twins of the environment, making it particularly useful in various fields such as factory robotics, autonomous vehicles, augmented reality, virtual reality, and geographic information systems (GIS) [12]. Fifth-generation cellular networks can be used for outdoor 3D mapping. Wireless fidelity (Wi-Fi) or Campus networks are used to enhance the existing indoor infrastructure map. Resource sharing can be done, particularly allocating specific carriers in orthogonal frequency-division multiplexing (OFDM)-based frames for sensing and preserving others for communication.

By using multiple sensors and communication channels, the system becomes more robust and resilient. Relying on data from different sources, the concept utilizes redundancy, reducing the risk of data loss and ensuring interactive map updates. The concept can be scaled to cover larger areas or complex environments. By adding more sensors and optimizing communication networks, the 3D map generation system can adapt to different scenarios and requirements.

#### 2.1.3. V2X Communication

V2X communication using JCRS is an emerging technology that enables vehicles to communicate with various objects in their environment, including other vehicles, pedestrians, and infrastructure to share the information collected from onboard sensors. This improves the road safety and traffic system resilience. V2X communication is crucial for autonomous vehicles. It allows for autonomous cars to share their intent with other road users, making their behavior more predictable and fostering a smoother integration with human-driven vehicles. Ref. [13] elaborated a system model and key parameters for JCRS in FR2 mmWave band. Resource sharing can be implemented by embedding one of existing modulation schemes into frequency modulated continuous wave (FMCW) waveforms, thus enabling low data rate communication between the vehicles. Sharing the relative position and velocity information of the neighboring objects will increase the situation awareness on a local scale. The same information communicated through a cellular network from other traffic entities (with a cyber presence) will be processed as well.

#### 2.1.4. Other Scenarios—Today and Tomorrow

The number of applications and services requiring JCRS are growing every day. Ref. [14] discussed the applications in both communication and radar sensing domains for different frequency bands. Sub-8 GHz bands are reserved for long-term evolution (LTE) and wireless local area network (WLAN) in the communication domain, whereas in these bands, many radar applications exist as well, e.g., moderate and long-range surveillance radar, air traffic control (ATC) radar, weather radar, and early warning radars. In the mmWave band, automotive and high-resolution imaging radars exist. In recent years, the potential usage of 60 GHz for IEEE 802.11ad/ay [15] communications has reduced the gap between the two systems, with front-end, antenna, and packaging technologies that can address the requirements for both systems. From a localization perspective [6], the needs of sensor fusion, robot localization, object recognition, context awareness, AR/VR/mixed reality (MR), and beam search in order to enable low power directional transmission have been caused an exponential increase in interest regarding joint communication and sensing, and specifically JCRS. 

### 2.2. Key Enablers and Driving Factors

The advancement of complementary metal-oxide semiconductor (CMOS) and silicon germanium (SiGe) Bipolar CMOS (BiCMOS) technologies have boosted the performance of RF front-ends in the last decade, thus making it possible to have low-cost consumer-ready solutions [15,16,17]. A big driving factor has been the spectrum crunch in sub-8 GHz bands. The 5G-NR bands at cmWave, the increasing interest in the 6–16 GHz bands [18] and available hardware at 60 GHz can be identified as the key enablers bringing JCRS systems to the consumer market. In the radar domain, range resolution depends on the usable bandwidth. The mm-scale range resolution is possible while using 60/77 GHz frequency bands. The higher frequencies, namely, 110–140 GHz bands [19], can also effectively enable new use cases in AR/VR and Industry 4.0 use cases. Innovative technologies, such as metasurfaces and intelligent reflective surfaces [20], add new dimensions. The new hardware technologies are discussed in Section 3. The trends of cellular networks going into 6G and beyond are discussed in [21]. One trend is to move into higher frequency bands that support a wider bandwidth. As the radar range solution becomes better with a higher bandwidth, this trend will facilitate JCRS systems greatly.

**Figure 2:**
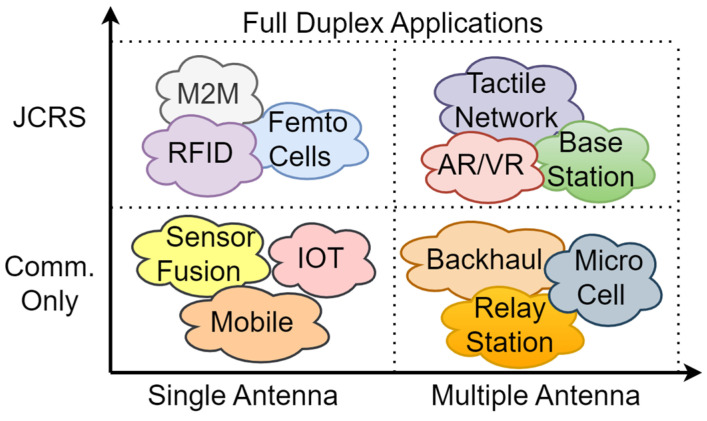
Application scenarios for in-band full duplex operations.

In-band full-duplex operation is also envisioned as one of the key enablers. It will enable better spectral re-use, adding minimal channel interference considering spatial diversity at mmWave frequencies [22]. Figure 2 depicts the classification of different applications in terms of cost and complexity, which will potentially operate in full-duplex mode. For applications such as IoT routers, a single antenna communication link will be sufficient, as it does not require a great cost. On the other hand, high-cost AR/VR applications require multiple-input and multiple-output (MIMO) capabilities with integrated sensing in order to enable real-time interaction with the additional realities. For some applications, such as machine-to-machine (M2M) or radio frequency identification (RFID), the classification border is vague; however, it helps to identify niche solutions for those.

## 3. Circuit Level Perspectives of JCRS RF Hardware

From an RF hardware perspective, the sensing and communication operating frequency bands were traditionally different until recently, when mmWave front-ends and the usage of bands above 20 GHz became reality in 5G networks. Still now, most of the consumer communications happen below 10 GHz and most commercial radars use E bands, namely, 77–81 GHz. Apart from the differences in frequencies, there are several other differences that can be noted, as given below [8,23]:

- Simplex/duplex operation modes—while most communication systems operate at simplex or frequency division duplex (FDD) or time division duplex (TDD), most radars utilize in-band full duplex operation. Respective scenarios are mentioned in Section 2.2.
- Transmitter to receiver isolation—Due to the different operation modes, transmitter to receiver leakage, and the resulting management thereof, self-interference cancellations have played different roles in communication and radar systems. Section 4.1 provides more details on this topic.
- Bandwidth requirements—As the center frequencies are different, the usable bandwidths in communication bands are limited, even though the fractional bandwidth values are similar for radar and communication operations. The absolute bandwidth requirements for radars are usually higher than those for communication systems because of the range resolution needs. Nevertheless, these resolution requirements will demand a higher fractional bandwidth if radar sensing is done at lower than 10 GHz frequencies.
- Dynamic range and linearity requirements—To support complex modulation schemes, communication systems require higher linearity than radars. At the same time, radar receivers need to detect weaker signals in the case of middle and long-range radars.
- Antenna requirements—For most traditional communication systems, wider beams are used, whereas the automotive radars use narrower beams to improve on the link margin at mmWave frequencies. Section 3.2 provides further details.
- MIMO and BF at mmWave frequencies—Differences exist even when similar frequency bands are used. The communication needs BF to be able to search receivers, as well as to boost the link margin. The radar traditionally has more receiver antenna elements than the transceiver antenna and it may not necessarily require BF. However, most radar systems are equipped with BF nowadays. Section 3.3 provides further information on this topic.

Apart from the design and deployment of hardware, the accurate modeling and inclusion of impairments in system simulations have been stressed [24,25]. In the below sections, different aspects of JCRS hardware developments are described, followed by packaging considerations. 

### 3.1. Front-Ends

As evident from the diversity of applications, no single front-end can fit all applications. Some use cases are limited by the area of the implementation as well as the number of front-ends/antennas that can be used, especially in the case of hand-held devices. Automotive applications use multiple radars to ensure reliability and are more performance oriented, whereas the base stations require optimization based on both power and performance. A comparative analysis of front-ends is given in [8,23]. 

**Figure 3:**
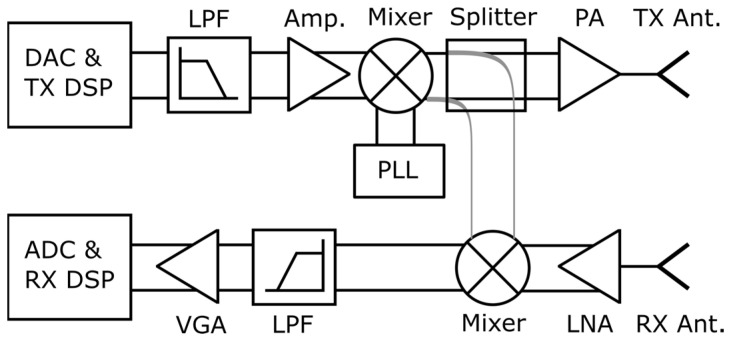
A representative radar front-end. DAC = digital-to-analog converter, LPF = low pass filter, Amp. = amplifier, PA = power amplifier, PLL = phase locked loop, TX Ant. = transmitter antenna, RX Ant. = receiver antenna, LNA = low noise amplifier, VGA = variable gain amplifier, ADC = analog-to-digital converter, DSP = digital signal processing.

**Figure 4:**
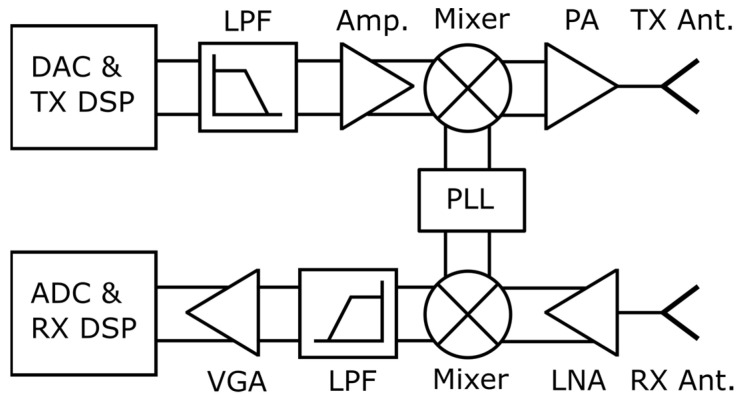
A representative communication front-end (short forms as defined in Figure 3).

Traditionally, front-end communication chips are already available from 24 to 71 GHz ranges thanks to 5G NR frequency range 2 (FR2). The use of mmWave frequencies in both communication and radars can be a key enabler for a combined front-end, even though, traditionally, front-ends are different, as shown in Figure 3 (radar) and Figure 4 (communications). However, it is important to note that many blocks such as mixers, power amplifier (PA), low noise amplifier (LNA), and baseband (BB) variable gain amplifier (VGA) are present in both. The main difference lies in the requirements of these blocks and in ADC (analog-to-digital converter)/receiver digital signal processing (RX DSP) blocks. Radars use mostly stretch processing using FMCW waveforms, which is enabled by analog signal processing and light weight basebands. On the other hand, communication front-ends use digital processing and more spectrally efficient waveforms such as OFDM, as shown in Figure 4. The gaps become thinner when it comes to using an OFDM radar, which uses the same waveform and requires more advanced DSP than the FMCW radar. However, compared with the communication counterparts, the baseband processing differences will remain, as the transmitted signal and timings are known in the radar use case. In communication use cases, the transmitter is located in another device. 

Some of the key performance indicators (KPIs) for a communication front-end are receiver gain and its ranges/controllability, receiver linearity, receiver noise figure, bandwidth of operation for both transmitter and receiver, PLL jitter/phase noise, power amplifier 1-dB and saturated power values, capability of duplex operations (e.g., simplex, half-duplex or in band full duplex), etc. To increase the spectral efficiency or bits/Hz, higher modulations schemes are being used in recent communications systems, thus necessitating the PA to operate at 6 dB or more backoff from the output 1 dB compression point (P1dB). The noise characteristics of different blocks also play an important role in high data throughput communication systems. On the other hand, radar KPIs primarily include bandwidth, receiver sensitivity, and transmitter saturated output power (PSat). While communications systems may operate at 100 MHz bandwidth, radar systems become severely limited in terms of range resolution for such a limited bandwidth (e.g., 1.5 m for 100 MHz bandwidth). A typical radar transmitter operates at the highest permissible power to increase the detection range in contrast with communication transmitters. However, in terms of LNA design, high gain and low-noise-figure LNAs [26] facilitate both the systems. For a chirp-based radar, PLL is used to create the frequency chirps, whereas in communication systems, after calibration, the frequency of PLL output stays constant as long as the used band is not changed. The authors of [23] outlined further details about these differences and the inherent similarities of different blocks. If communication and radar systems have the same standard specifications in future networks, the gaps will reduce further. More details about the power, sensitivity, and operation modes are given in Section 4.

**Figure 5:**
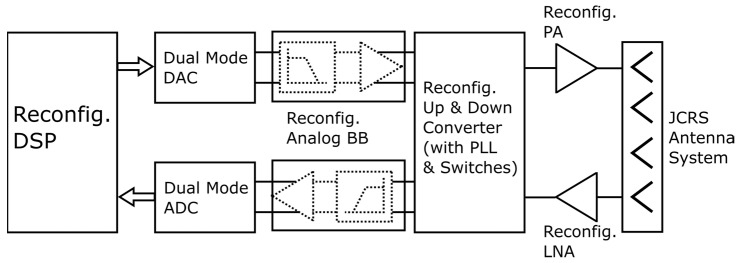
A representative reconfigurable JCRS front-end (short forms as defined in Figure 3).

In terms of front-end architecture, previous works [27,28,29] showed initial promising results, even though a unified framework for joint hardware usage will require a crisper definition of applications and related KPIs. Ref. [27] utilized a FMCW radar front-end focusing primarily on radar KPIs and utilized the same radar for communication operations. Ref. [28] used a common front-end with a novel radar-communication modulation scheme. Ref. [29] proposed a reconfigurable approach with a switched architecture. The front-end can act as a radar or communication front-end depending on the inputs to the receiver mixer from the transmitted signal via a splitter or directly from PLL. Such a reconfigurable architecture could be a key to hardware reuse if the individual blocks can also be made dual/multi-mode. Such an architecture is shown in Figure 5. However, additional switches or the inclusion of programmability may impact the performance, power, and area for the front-end chains, necessitating new ideas to build reconfigurable JCRS front-ends with impacting operations.

Apart from the differences in working principles, there are differences in technology for communication and radar chipsets. In the last few decades, several communication systems have moved to complementary metal oxide semiconductors (CMOS) for the implementation of front-ends to address the baseband processing needs. Apart from bulk CMOS processes, in recent years, there have been significant development in fully depleted silicon-on-insulator (FDSOI) processes [30]. III-V semiconductor processes such as GaAs for realizing the power amplifiers are still used but have been replaced by CMOS power amplifiers in recent years. However, given the output power requirements and relatively simpler baseband processing needs, radar chipsets are not limited to CMOS. They can use GaAs process or Silicon Germanium (SiGe) heterojunction bipolar transistor (HBT) processes. A recent work [31] showed promising results in GaN for mmWave operations of radars and communications. However, cost plays a role in the choice of technology, while considering mass volume production. However, these gaps also reduce depending on whether on-package multi-chip architecture is used at mmWave with front-ends in one process and baseband in another process, as mentioned in [32]. 

The good news is that the front-end hardware will not be a bottleneck, as in the last decade, there has been a significant development in front-ends and BFs to support mmWave bands by academia and industry. For instance, wireless backhaul applications utilize the mmWave spectrum for communications. Similarly, 60 GHz bands have already been used for gesture recognition [33,34]. The 60 GHz bands already have communication front-ends with BF capabilities [15]. Beyond 60 GHz bands, the 110–140 GHz frequency range [19] has also been identified for potential JCRS hardware. There are already several academic and industrial works in this range for radar [16,35] and communications [36,37,38]. The potential usage of advanced nodes of CMOS (40 nm or below) for mmWave applications [17] and other processes for high performance devices (e.g., SiGe BiCMOS [16]) will ease the realization of low-cost JCRS hardware front-ends.

There will still be challenges to solve in order to create a compact power-efficient hardware to support JCRS applications that are intended to enable tactile internet [39] and future consumer needs. In addition, antenna isolation may play an important role with reconfigurable front-ends in order to manage self-interference cancellation (SIC). The methods to achieve antenna isolation are covered in Section 3.2.

### 3.2. Antenna Implementation

The different antennas that are used for radars and communications are discussed below, with a comparative analysis for JCRS usage in Section 3.2.3.

#### 3.2.1. Antennas for Radar

The characteristics of EM radiation allow for the range to be measured using radars [40]. When EM waves collide with an electrically conductive surface, they are reflected. The presence of an obstruction in the propagation path is indicated in the event these reflected waves are subsequently received at the source. In air, EM radiation moves at a constant speed that is comparable to the speed of light. The preset velocity enables the calculation of the distance amid the radar system, reflecting objects such as aircraft, ships, or automobiles by formulating the elapsed interval for the broadcast signals. Antennas constitute the most important components of the radar system owing to their ability to transmit EM waves to spaceborne signals with the requisite efficiency and to ensure that the signal maintains an appropriate spatial pattern. Additionally, antennas are in charge of providing target position updates at the desired frequency, while offering the pointing direction with the utmost accuracy. Hence, the fundamental performance of the radar system may be demonstrated to be proportional to the product of the antenna extent and the median power being transmitted. Recent developments in the mmWave technology have led to the establishment of trustworthy radars for a variety of applications; for instance, preventing obstacles, landing assistance, autonomous vehicle control, accident avoidance, roadway safety aids, weather surveillance, mapping, healthcare diagnostics, and many more [41,42,43]. The commercialization of mmWave radars is primarily attributed to several significant advantages that mmWaves have over lower frequency bands. Radars that operate in the mmWave range are more compact and lightweight [44]. In particular, antenna dimensions and weight can be greatly decreased, while maintaining an identical beam width and gaining performance. Single-element construction, which may include planar, metasurface, transparent material, slot, defected ground, and other types, represents relatively simple and compact mmWave antennas. However, in terms of bandwidth, gain, and efficiency, the single-element configuration performs unfavorably. Additionally, the radiation pattern is mainly omnidirectional, whereas radar applications necessitate a directed radiation pattern. These challenges can be solved by array antennas as they offer numerous benefits such as adaptive BF, wide-angle beam scanning, multiple beams, increased gain, and wide bandwidth. 

Generally, two modes of operation exist for radar antennas, which include scanning and tracking. All of the objects in a segment are illuminated at regular intervals when the scanning mode is active. The antenna is devoted to one particular target and automatically monitors it while it switches orientation in the tracking mode. The capacity of the antenna to accurately determine the angle error is imperative to enable automatic tracking. The angle error indicates the angle between the antenna beam’s peak and the target’s direction. A high signal-to-noise ratio allows for precise determination to barely any of the beam width. Two configurations—the parabolic dish and the phased array—have been traditionally used to accomplish the functions of radar antennas. A summary of their underlying concepts is provided below.

Parabolic reflector: A dish antenna is made up of a parabolic reflector and a point source feed positioned in its center [45]. The innermost portion of a metal mesh-covered frame often makes up the construction of the parabolic reflector. The metallic covering serves as the reflector for the radar energy.

MIMO Antennas: In comparison with single-element antennas, array antennas offer better bandwidth, gain, and radiation performance; however, there has been no significant advancement in the performance of radar systems in dense environments where line of sight communication is impracticable. Hence, radar systems composed of MIMO antennas at the mmWave band are anticipated to accomplish high spatial resolution, optimized signal-to-noise ratio, decreased susceptibility to interference, enhanced positioning, and target discovery in comparison with conventional approaches [14]. A MIMO system includes several transmit antennas, each of which transmits an arbitrary waveform independently of the others. These signals may be received by each receiving antenna. Two sorts of MIMO radar systems are discernible [46]. The first is the monostatic MIMO radar, which employs collocated antennas and uses point targets similar to conventional radar systems. The second type of MIMO radar is distributed or bistatic and contains widely spaced antennas, each with a distinctive perspective of the target. The following presents a synopsis of their operating principles.

Monostatic radar: The antenna elements are well-positioned to one another to detect indistinguishable target radar cross sections (RCS). As with a phased-array antenna, each radiator in this setup features an independent transceiver module and analog-to-digital converter. A phased-array antenna, on the other hand, simply transmits an impersonation of a transmission signal generated in the central waveform generator through each radiator. As each radiator in a MIMO radar system features its individual arbitrary waveform generator, every radiator produces a distinct waveform. The identification of the echo signal’s source is also based on this particular waveform.

Bistatic radar: Each antenna views the target through a distinct angle, in contrast with monostatic MIMO. As a result, every antenna on the target has a different RCS. Analyzing radar data on such targets necessitates far more intricate target models.

#### 3.2.2. Antennas for Communications

An effective MIMO system requires that the antennas are isolated from one another. The performances of antennas and propagation channels, including the radiation pattern, gain, side-lobe level, channel capacity, and impedance matching have been determined to be severely affected by the mutual coupling between MIMO antennas at the mmWave band. Typically, an antenna isolation strategy involves maintaining an inter-element distance over half of the free-space wavelength. However, an enormous board space is needed for this approach. For various frequency bands over the years, a number of techniques have been investigated to improve MIMO antenna isolation, but very few techniques have been investigated for the mmWave frequency band. Planar MIMO antennas operating in the mmWave spectrum can be classified based on their structural configuration, as slotted radiator, defected ground, and metasurface [47]. Waveguide represents another important classification. 

Slotted radiator: The majority of planar antennas are created on a single substrate, which lowers their fabrication complexity and minimizes their costs. Slots are incorporated into planar antennas to lower mutual coupling. In the radiating plane, slots typically have the following shapes: tapering [48], fractal [49], coplanar waveguide [50], etc. The main goal of the method is to avoid requirements for a decoupling framework by concentrating the highest current near the slits, which lowers the mutual coupling between MIMO antennas.

Defected ground: The ground plane in planar antennas serves as a reflector, enabling EM waves to be normal to the radiating element. However, the ground plane may be adjusted from full to only a portion, known as defective ground structures (DGS), to accomplish better isolation between MIMO units [51]. In theory, defects on the ground plane interrupt the ground plane’s current distribution. This perturbation modifies the properties of a radiating structure by adding some slot parameters to the transmission structure parameters, such as resistance, capacitance, and inductance. DGS may be etched into any geometric shape, such as a circle, rectangle, and ring, to improve the targeted parameter.

Metasurface: The term “metamaterial” refers to a material that has been manufactured artificially with the intention of lowering the permeability, permittivity, and refractive index values [52]. When applied to the rear of the radiating device, a metamaterial construction with an EM band gap functions as a reflector to improve the radiation pattern on the broadside [53]. A “metasurface” signifies what is used to describe a metamaterial that has been etched on the same plane as an antenna element. Better isolation among radiating elements may be achieved by using a metasurface layer along the radiating element.

Waveguide: Waveguides feature extremely low transmission losses in the mmWave spectrum in contrast with losses via printed transmission lines [54,55]. The application of rectangular waveguides provides an extensive amount of flexibility in the choice and fabrication of antennas, including horn antennas [56], waveguide radiators [57], and wide-scan waveguide radiators [58]. Waveguides additionally exhibit remarkable isolation characteristics.

#### 3.2.3. A Comparative Analysis and Steps for JCRS Antenna Enablement

Numerous antennas, for both radar and communications, have been reported over the years. Similar design principles have been used in both applications. A comparison has been made between a few recently presented antenna types that can be used for radar and communications. The comparison is summarized in Table 1.

The performance of a parabolic dish reflector-based antenna is good, but the milling and manufacturing of such antennas have a high production cost and labor-intensive procedure. Planar arrangements are thus a good choice, because they have relatively low production costs and quick processing. By utilizing various techniques, the desired antenna performance can be determined from planar layouts. Typically, slots are added to the ground plane or radiator to achieve a wide band performance, but with limited isolation properties. Waveguide-based antennas can produce high isolation characteristics, but at the expense of bandwidth. In order to meet the demands of the upcoming generations of communication standards, researching several MIMO antenna isolation augmentation approaches at the mmWave spectrum is necessary. In particular, different metasurface arrangements must be studied to attain EM properties that are unattainable with natural materials.

### 3.3. Phased Arrays and Beam Steering Systems

BF is intended for electronically steering the beam pattern of an antenna system. Conventional radars already use BF for spatial filtering, while conventional communication systems prefer a wide and stable antenna pattern for coverage. However, the recent exploitation of the mmWave spectrum has forced communication systems to adapt to high gain antenna systems for counterbalancing the path loss, resulting in narrow beam utilization supported by BF for coverage.

The exploitation of the mmWave spectrum has reduced the difference between antenna systems for radars and communication systems because BF has become a must for both; thus, the chances of JCRS are enhanced. In a broad categorization, a beam steering system is comprised of a phased antenna array and a BF network. Both sub-systems can be further classified into several categories and are independent of each other to a large extent, with respect to their design procedure and goals. 

#### 3.3.1. Phased Arrays

An antenna array is comprised of several individual antenna elements assembled in a linear or bi-linear configuration. When each element is excited with a controlled phase shift with respect to the neighboring element, a variation in beam angle can be achieved in the space. Active return loss may result when several elements are excited simultaneously due to their mutual coupling. Mutual coupling depends on the beam width, bandwidth, and element type. A high return loss may cause scan blindness for targets and loss of coverage in communications. Therefore, a single element may require redesigning or tuning for operation in an array due to pattern multiplication. Mutual coupling can be reduced by increasing the inter-element distance, at the cost of strong side lobes appearing around the main lobe, called grating lobes. Grating lobes can be avoided by limiting the interelement spacing *d* as [66]:

(1) $$d\;⪅\frac{\lambda }{1+\left|\sin\left(\theta_{max}\right)\right|}$$

where *λ* is the non-guided wavelength and *θ* is the beam steering angle from array symmetry axis. To achieve a bore sight to end-fire beam steering, *d* should be less than half the wavelength at the highest frequency of operation. It gives rise to strong mutual coupling with ordinary wide patch-antennas. Therefore, compact element designs are required for achieving tight inter-element spacing. Grating lobes are less vital for radar-assisted communication compared with radars as a primary service application where spatial accuracy of the target is of higher importance.

In addition to grating lobes, gain fluctuations arise from the array gain dependence on the beam angle and frequency-dependent pattern of individual elements [67,68]. Gain fluctuations are relatively more critical to radar systems than communication due to the increased probability of target missing in the low-gain conditions. Gain fluctuations can be compensated on the penalty of complex signal processing.

#### 3.3.2. Beamformer Networks

The relevant phase for each element in the array is generated through a BF network. The requirements for the BF network can be separate for communication and radar systems, e.g., beam switching angle, switching time, signal processing needs, and reciprocity of the network. Its application in both systems simultaneously depends on the target application. In this paper, we classify the BFs broadly in term of circuit/hardware implementation as active and passive networks.

Active BFs, when implemented in the analog domain, have the mostly widely adopted and simple implementation. Each antenna element is fed through a dedicated phase shifter either placed in an RF or local oscillator (LO) path, also known as an RF or LO path BF [69]. On the other hand, active digital beamforming exploits signal processing capabilities to process a unique stream for each antenna element with the required phase shift. It provides maximum control over beam patterns and calibration compared with all of the other implementations. However, it requires an increased number of digital-to-analog converters (DACs) or analog-to-digital converters (ADCs) and RF chains. Another approach is hybrid BF [70], by connecting several antennas to a limited number of ADCs/DACs through analog beamformers. It provides an extended beam control compared with pure analog BF, with reduced digital processing compared with pure digital BF [71]. Full duplex JCRS has been reported using such a BF in [72]. Active BF requires separate phased arrays for the transmitter and receiver because of the unidirectional behavior of active components and DACs/ADCs, which not only increase the size of the antenna system, but also disturb roundtrip channel reciprocity. At miniaturized wavelengths, the transmitter-to-receiver array center-to-center distance becomes greater than half the wavelength. In this case, the transmitting and receiving waves adopt different paths in a multipath environment. This phenomenon is unfavorable for several communication applications, depending on round trip channel reciprocity, e.g., physical layer security [73]. However, it has no operational downside for monostatic radars. 

On the other hand, passive BFs are relatively large structures implemented off-chip. Lens-based passive BFs are based on optical technologies. A dielectric lens is produced through printed circuit technology (PCT) and the radiating part is connected to the antenna array e.g., Rotman lens [74] and Ruze lens [75]. An alternative implementation is a circuit connecting each input of the network to each antenna element, such that a unique output phase is generated by exciting the network from each input. The popular implementation is Butler matrix, comprising of fixed phase shifters and quadrature hybrid couplers [76,77].

In addition to PCT, the Butler matrix has been reported in waveguide and CMOS technologies [78]. Passive BFs work on true time delay principles and provide a frequency-dependent phase shift over a bandwidth, unlike active phase shifters. Hence, they do not cause beam squint, which is the frequency dependency of beam angles notable in active analog BFs. Beam squint is not a problem in narrow band communication and has been utilized in wideband communication as a frequency scanning array [79]. However, wideband narrow-beam radars can experience reduced returns due to this phenomenon. Since true time delay is not possible on-chip due to the extended required area, and off-chip passive BF provides a few discrete beam angels, digital BF can be attractive for high resolution radars based on true time delay in the digital domain. Another advantage passive BFs have is their reciprocity. A single antenna system can be used for transmission and reception as phased arrays are already reciprocal. It not only reduces the form factor, but ensures channel reciprocity in mmWave bands. A useful application of the Butler matrix is given in [77] for full-duplex communication with monostatic radars in a JCRS scenario. 

A fully digital beamformer at 140 GHz has been presented in [80] using 128 element phased array. Since digital beamforming is not always practical due to its high power consumption, analog beamforming can be attractive above 100 GHz due to the reduced circuit size. However, BFs above 100 GHz become challenging due to phase shifters’ higher loss and noise figures. BF requires RF blocks with controlled phase variations, which also become difficult due to parasitics at this frequency. One potential solution can be high IF BF using IF frequencies between 10–20 GHz, as reported in [81].

### 3.4. Packaging Considerations

Packaging of semiconductor circuits becomes critical with the increase in operational frequencies. The chip-to-board interconnect exhibits strong parasitics, affecting the system performance for mmWave carriers. Bondwires have been a popular choice for interconnects, which show frequency and length dependent inductance, making a bottleneck between the chip and carrier. Nevertheless, it has been extensively used in mmWave circuits with various parasitic compensation techniques, e.g., tight ground-signal-ground (GSG) configuration, half-wavelength, and planar lumped element matching [82,83]. Matched bondwire interconnects have been used for operations above 100 GHz [84]. The alternative is the flipchip approach, offering very short length chip-to-carrier connections. The downsides of flipchip are complex placement, dielectric detuning, and restricted heat relief paths. 

Besides interconnects, array feed networks require customized passive RF blocks. Surface mount lumped components are typically not functional beyond the cmWave spectrum. Hence, distributed solutions are preferred for baluns, couplers, and bias-feeds, e.g., with antenna switches and phase shifters. PCT has been widely adopted in electronic packaging and antenna design. The first-class manufacturing facilities can offer multilayer packaging with design resolutions down to 70 μm. The process tolerance of PCT is usually higher than thick and thin film processes. and becomes a point of concern as the wavelength shrinks. In contrast, advanced integration technologies, e.g., co-fired ceramics, quartz, and silicon interposers, can offer layout resolution of a few micrometers and an excellent accuracy, with the penalty of fabrication cost.

The advancements in chip-to-carrier interconnect and planar patch antenna designs have enabled antenna-in-package (AiP) possibilities, easing interconnections and economical manufacturing. mmWave frequencies are favorable for AiP, offering compact form factors. A challenge in densely packed antenna packages is RF-level transmitter-to-receiver leakage, potentially saturating the receiver low noise amplifier (LNA). Various approaches are being research for transmitter-to-receiver isolation enhancement, namely DGS, EM bandgap assemblies (EBG), and interleaved antennas [51,85]. Mitigating the transmitter leakage can enable JCRS implementation with antenna system sharing between in-band full-duplex communication and monostatic radars. 

At D-band, this becomes more challenging. The bondwire interconnects have frequency-dependent loss, and they are not preferable at D-band. Flipchips offer very short length interconnects and are still usable. Ref. [86] presented a prototype with a flipchip interconnect on an organic interposer containing the antenna, which is then bonded to PCB using ball grid arrays. Ref. [81] presented a packaging approach using EM coupled interconnects. A diced quartz with a printed antenna is attached to the top of chip. An on-chip feed line couples a 140 GHz signal to the patch antenna on the quartz superstrate. The chip-quartz packaging is then bonded to the PCB using bondwires for IF and LO ports. Other packaging options include fan-out wafer level packaging, used in [87], for hybrid beamforming in communication and localization.

## 4. System Level Perspectives of JCRS RF Hardware

The critical system perspectives are described in the following sub-sections, which have not been covered in earlier sections. 

### 4.1. Full-Duplex and Self-Interference Cancellation

Full-duplex operation is essential in dense networks, where spectral efficiency will be an important key performance indicator (KPI). However, self-interference cancellation is the major obstacle for enabling full duplex operation, particularly for monostatic operation in V2X modules and baseband cellular station.

**Figure 6:**
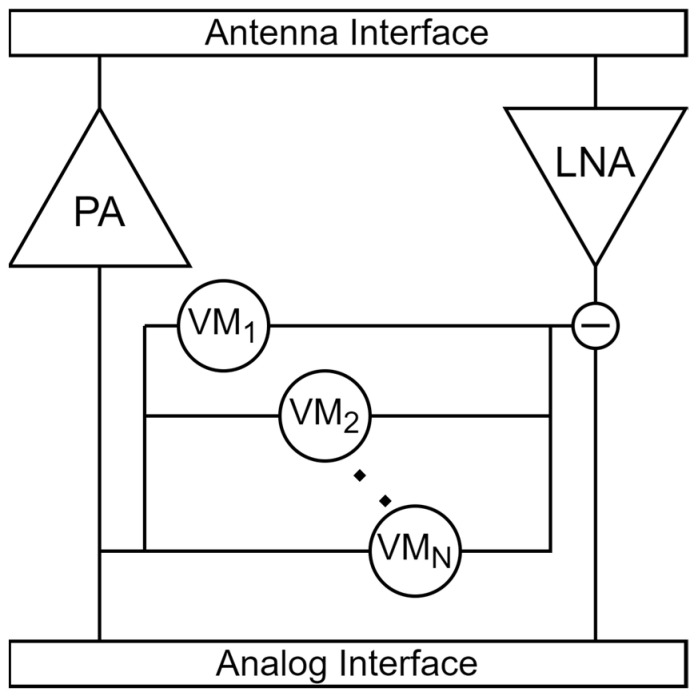
RF Self Interference Cancellation.

Self-interference cancellation is essential in enabling full-duplex operation. The transmitter signal, which leaks to the receiver chain through different mediums, overlaps the received signal. It can be cancelled in different domains. Antenna isolation/cancellation techniques have been already covered in previous sections. The leaked signal can also be cancelled in RF, analog, or digital domains. Figure 6 shows the cancellation scheme in the RF domain. Multiple taps are used to modulate the known transmitter signal and subtract from the received signal, thus filtering the received signal. In practice, all three domains are deployed, thus reducing the dynamic range requirements for each stage. Self-interference in the digital domain exhibits a higher cancellation capability as the non-linear components and phase equalization are considered. Dynamic range requirements are dependent on both external and internal specifications, such as bandwidth, hardware non-idealities, and digital signal processing gain.

### 4.2. Link Level Perspective for Application Scenarios

For V2V applications, communication-assisted radar functionality can be implemented by embedding one of the existing modulation schemes into FMCW waveforms, thus, enabling low-data-rate communication between the vehicles. For example, quadrature amplitude modulation (QAM) can be implemented by modulating the conducted power and phase of FMCW waveforms. As the phase information is used for velocity estimation, communication phase de-embedding is used.

When it comes to digital twining, radar-assisted communication reuses the existing infrastructure, which is based on the OFDM modulations scheme. The whole available bandwidth can be used for sensing and increasing the range resolution. However, this will reduce the sensing signal-to-noise ratio. Recent approaches allocate a number of subcarriers for sensing, maintaining others for communication [13].

**Figure 7:**
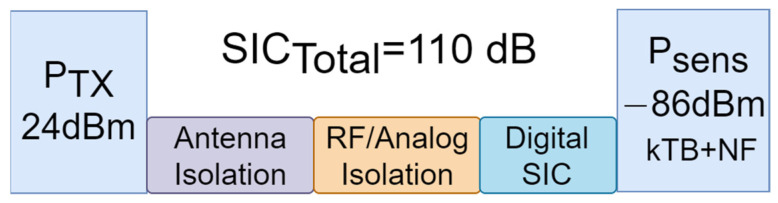
Power link budget. (k—Boltzmann’s Constant, T—Temperature, B—Bandwidth, NF—Noise Figure; NF = 8 dB assumed, so the sensitivity level Psens = kTB + NF = −86 dBm).

For both FMCW and OFDM modulation schemes, self-interference cancellation is crucial to suppress the transmitter signal leaked into the receiver chain. The conducted power (P_TX_) is normally regulatory limited, i.e., 20–24 dBm for the transmitter conducted power [88]. Ideally, we need to bring this below the noise floor, which is defined by the channel bandwidth. More isolation may be required in the case of substantial signal processing gain. Figure 7 shows a use case for a communication/sensing channel with 100 MHz bandwidth. As can be seen, 110 dB total isolation is needed to fully suppress the leaked signal. It is worth noting that, with increasing the bandwidth, the isolation requirement could be potentially relaxed, as P_sens_ increases with that.

### 4.3. Beyond the Hardware

As mentioned in the previous sub-sections, the intended waveforms and related signal processing play an important role in the use of the hardware. Waveforms are by far the most investigated topic in this field, considering different high energy efficiency and high spectral efficiency options [89,90]. A reconfigurable and flexible physical layer will definitely help to balance out any over-design requirements. Ref. [91] proposed a multi-functional receiver to facilitate the spectral convergence. In the medium access protocol (MAC) layer, new algorithms, specifically machine learning/artificial intelligence-aided algorithms need to be integrated. The networking layer needs to account for the additional latency and security requirements in critical applications. The security aspects while using beamforming will be of paramount importance [92]. Latency will be important for RaaS as well. New networking concepts for 6G integrating IoT devices need to be considered [93]. The possibilities will also be determined by the standardization activities. The overall software and operating systems supporting the devices using JCRS need to account for additional privacy or security requirements of data collection and usage. 

### 4.4. System Validation

Testbeds to validate the use cases also play an important role to identify additional use case bottlenecks. The use of software-defined radio platforms can facilitate the researchers in the following three steps:

- A use case and new waveform/signal processing validation platform using standard available RF front-end and available antenna structures [94].
- Replacing the antenna with a custom antenna system with enhanced isolation or enhanced bandwidth.
- Replacing the standard front-end with a custom JCRS front-end with enhanced isolated antenna system.
- In addition, there are different demonstrations of JCRS use cases, available in the literature. The authors of [91] considered a JCRS spectral convergence scenario for collision avoidance in an intersection. The urban connected car scenario is used in [95] when the bandwidth was limited due to sharing with communication services. A framework of using 802.11ad compliant radar is described in [96]. Ref. [97] discussed Wi-Fi sensing use cases in details.

## 5. Discussions and Conclusions

There have been several research studies for JCRS in the past, with a focus on waveforms and network possibilities [1,2,4,5,8,14]. However, a comprehensive survey on the RF hardware comprising of front-ends, antenna, beamformers, and packaging along with the related system perspectives has been missing in the literature. This article aims to fill that gap, while focusing more on the mmWave implementation of such systems. 

JCRS is a power technology, which will increase the resilience and security of almost all verticals. However, much research is still ahead, especially for full-duplex operation. To design compact RF hardware, achieving high bandwidth and self-interference cancellation will be the next milestone in enabling high throughput and high resolution JCRS systems for future 5G/6G ecosystems. However, depending on the figure-of-merits, the nature of integration will vary. The introduction of the 802.11bf standard [98] for Wi-Fi sensing will play an important role as a starting point. The high bandwidth available in ultra-wide band (UWB) standards can play a role as UWB sensing to support presence detection and environment mapping. The first generations of JCRS systems may have only minor modifications over the traditional RF hardware, mostly due to the missing demand from the users. However, as discussed in this paper, the path to solve the challenges for a co-designed system, optimized for hardware reuse, needs to be paved without additional power and area overheads. The main research activities should focus on the realizability of a low power compact mmWave solution that can address KPIs for both communication and sensing. The benefits are limited if the two systems are working only in cooperation. Hardware reuse and a co-designed reconfigurable RF hardware will provide the right impetus towards making RaaS a reality. The opportunities are limitless, whether it is the coordination of radars in the context of V2X or to build context aware communication systems enabling 6G networks. 

Standardization process will be another milestone in reaching spectrum sharing, synchronization, localization, and other KPIs such as the other systems of radar-assisted communication, communication assisted radar, Radar as a Service, etc. The effort will ensure the seamless co-existence of communication and radar sensing systems, bringing more resilience for smart cities, smart factories, and other verticals. The success of 802.11bf systems will also a play a role in understanding the first usage of integrated communication and radar sensing using the same band. However, it is of paramount importance to verify the possible hardware architectures for JCRS systems as inputs to the standardization and case study discussions in order to facilitate the integration of this powerful feature/technology in 6G and beyond communication networks. 

## Figures and Tables

**Table 1:** Comparison between different radar and communication antennas.

Application	Antenna Type	Frequency (GHz)	Isolation (dB)	Reference
Radar	Parabolic reflector	20; 40	---	[45]
	Waveguide + two stacked printed circuit boards (PCBs) + metallic vias + linear array	31–41.5	12	[59]
	Slotted radiator (Vivaldi)	8.3–11	---	[60]
	Slotted radiator (rectangular loop)	1.5–3.5	---	[61]
	Slotted radiator (coplanar waveguide)	130–150	---	[62]
	Waveguide	76–81	30	[57]
Communication	Parabolic reflector	26.5–40	---	[63]
	Magnetoelectric dipole + phased array	26.5–29.5; 24.25–27.5	---	[64]
	Slotted radiator (tapering)	2.4–5.1; 5.1–5.6; 23–30	16	[48]
	Slotted radiator (fractal)	2.7–5.1	---	[49]
	Slotted radiator (coplanar waveguide)	1.3–40	22	[50]
	Metasurface (EM band gap)	54–65	30	[53]
	Waveguide	132–152	---	[65]

## Data Availability

No new data were created or analyzed in this study. Data sharing is not applicable to this article.
